# Investigating the Acceptance and Implementation Conditions of Telerehabilitation in Germany Among Patients and Health Care Professionals: Qualitative Interview Study

**DOI:** 10.2196/68766

**Published:** 2025-08-01

**Authors:** Monica-Diana Podar, Susanne Stampa, Oliver Razum, Christoph Dockweiler

**Affiliations:** 1 Department of Epidemiology and International Public Health School of Public Health Bielefeld University Bielefeld Germany; 2 Department of Social Sciences, Chair of Digital Public Health Faculty of Arts and Humanities University of Siegen Siegen Germany

**Keywords:** acceptance, barriers, Consolidated Framework for Implementation Research, CFIR, implementation, qualitative research, telerehabilitation, extended unified theory of acceptance and use of technology, UTAUT2

## Abstract

**Background:**

Telerehabilitation has become increasingly important worldwide, as the COVID-19 pandemic forced many rehabilitation centers to change their daily care routine and find new ways to provide medical rehabilitation and aftercare.

**Objective:**

This study aims to investigate the acceptance and implementation conditions of telerehabilitation in Germany, particularly following the COVID-19 pandemic.

**Methods:**

We conducted qualitative semistructured interviews with patients (n=9) and health care professionals (n=8) between September 2023 and January 2024. To explore individual and structural barriers to and facilitators of telerehabilitation adoption, we used the extended unified theory of acceptance and use of technology and the Consolidated Framework for Implementation Research.

**Results:**

Patients and health care professionals perceived telerehabilitation as positive, mainly due to its flexibility and accessibility. Patients expressed high acceptance levels, anticipating health benefits, although they found it challenging to familiarize themselves with the technology and establish routines. Health care professionals highlighted the need for adequate resources (financial, time, and personnel) and management support to implement telerehabilitation successfully. Both groups saw higher acceptance and cost coverage of telerehabilitation services as essential for successful implementation and use in Germany.

**Conclusions:**

This study identified institutional barriers, such as concerns about resource availability, team communication, and initial resistance among health care staff to the introduction of new technologies. At an individual level, we found that patients struggled with routine establishment and that digital and in-person support from institutions and peers could mitigate this challenge. Implementing a hybrid approach and improving funding and approval processes would enhance telerehabilitation integration in the German health care sector.

## Introduction

### Background

Telerehabilitation has become increasingly important worldwide, as the COVID-19 pandemic forced many rehabilitation centers to find new ways to provide medical rehabilitation and aftercare [1-3]. In this study, telerehabilitation is defined as medical rehabilitation services using information and communication technologies (ie, assessment, monitoring, prevention, intervention, supervision, education, consultations, and counseling) [4]. Telerehabilitation is used to complement or extend, but not to replace, face-to-face medical rehabilitation and aftercare. Benefits of telerehabilitation include flexibility in terms of time, location, and convenience. This is particularly advantageous for patients who are immobile or with comorbidities or those living in remote areas with poor health care infrastructure [5]. Previous research has mainly focused on demonstrating the effectiveness of standard care [6-8], with less attention given to issues such as the implementation process and acceptance [2].

Insight into the acceptance of telerehabilitation in different settings is critical as it serves as a prerequisite for using new technologies [9]. To the best of our knowledge, this is the first study that examines the acceptance and implementation conditions of telerehabilitation in the German rehabilitation context across different diagnoses since its introduction.

### Aim of the Study

The aim of this study was to investigate the acceptance of telerehabilitation among patients undergoing rehabilitation and to evaluate the implementation conditions from the perspective of health care professionals.

## Methods

### Design

This qualitative study was part of a larger explanatory mixed methods project examining acceptance and implementation of telerehabilitation in the context of the COVID-19 pandemic. Our goal was to contextualize the results of our quantitative survey findings [10] and gain deeper insight into the acceptance of and implementation conditions required for telerehabilitation among patients and health care professionals.

### Underlying Theories

This study focused on patient acceptance and the conditions for implementing telerehabilitation in Germany. To better understand the needs and experiences of the 2 stakeholder groups, this study was anchored in 2 theoretical frameworks.

For deeper insight and comparability, we anchored the interviews in the same frameworks used in the quantitative surveys with patients and health care professionals.

The extended unified theory of acceptance and use of technology (UTAUT2) [11,12] served as the theoretical basis for the patient interviews. The framework describes the different factors that may influence the acceptance and use of telerehabilitation, such as *effort expectancy, performance expectancy, social influence, facilitating conditions, hedonic motivation, habit, and price value* [12]. *Privacy concerns* were included in our study, as previous research has shown that this issue can play a role in the use of digital services, such as in the field of telemedicine [13,14]. We excluded *price value* from our interview guideline, as telerehabilitation is typically covered by Deutsche Rentenversicherung (DRV [the German Pension Insurance Association]) and is free of charge for patients (refer to Table 1 for definitions). In addition, the patient interviews also focused on general experiences and expectations of telerehabilitation use.

**Table 1:** Adapted extended unified theory of acceptance and use of technology (UTAUT2) dimensions included in the interview guideline.

UTAUT2 dimension [11,12]	Definition (adapted to the context of this study)
Effort expectancy	This reflects the degree of ease associated with the use of telerehabilitation.
Performance expectancy	It is the degree to which an individual believes that using telerehabilitation will help them.
Social influence	This is the degree to which a person perceives that people who are important to them believe that they should use telerehabilitation.
Facilitating conditions	This dimension reflects the degree to which an individual believes that an organizational and technical infrastructure exists to support the use of telerehabilitation.
Hedonic motivation	This reflects the fun or pleasure derived from using telerehabilitation.
Experience and habit	Experience reflects an opportunity to use similar telerehabilitation offers and is typically operationalized as the passage of time from an individual’s initial use of a technological system. Habit is the extent to which people tend to act automatically because of learning.
Privacy concerns (adapted from the studies by Zhou [13] and Smith et al [15])	These reflect fear that personal data will not be adequately protected.

The Consolidated Framework for Implementation Research (CFIR) served as the theoretical basis for the interviews with health care professionals to facilitate a better understanding of the institutional factors related to telerehabilitation implementation. The framework maps barriers and facilitators across 5 domains: *innovation, inner setting, outer setting, individuals,* and *implementation process.* We adapted the subconstructs to fit the study purpose and context in line with the CFIR recommendations [16] (Table 2 presents the CFIR framework with the 5 main dimensions and the subconstructs in this study). To identify the subconstructs relevant to our interviews, we built upon our previous quantitative survey of health care professionals. For the questionnaire, we identified the relevant subconstructs based on existing studies on the promoting and hindering factors of telerehabilitation and eHealth implementation [9,17,18]. Finally, the identified subconstructs were then discussed with the project advisory board, whose members consisted of experts from rehabilitation science and health care practice.

**Table 2:** Adapted and applied Consolidated Framework for Implementation Research (CFIR) dimensions included in the interview guideline.

CFIR domain	Definition (according to Damschroder et al [19])	Subconstructs of the dimension used in this study
Innovation domain	This domain discusses what is being implemented (eg, a new clinical treatment, educational program, or city service).	Relative advantages of the innovation Costs (eg, initial costs and monthly license fees)
Inner setting domain	This domain is defined as the setting in which the innovation is implemented (eg, hospital, school, and city). There may be multiple inner settings and multiple levels within the inner setting (eg, unit, classroom, and team).	Available resources (eg, space, personnel, and time) Communication (eg, information sharing practices) Organizational culture (eg, values, beliefs, and norms) Access to knowledge and information (eg, training programs)
Implementation process domain	This domain discusses the activities and strategies used to implement the innovation.	Assessing needs Engaging patients and health care professionals
Individuals domain	This includes the roles and characteristics of individuals involved in the implementation process.	Capability (eg, necessary skills and knowledge) Motivation Implementation leads and teams Support
Outer setting domain	This is defined as the environment in which the inner setting exists (eg, hospital system, school district, and state). There may be multiple outer settings and multiple levels within the outer setting (eg, community, system, and state).	Critical incidents as driving factors (eg, COVID-19) Policies and laws (eg, funding regulations)

### Participant Recruitment and Data Collection

Two researchers (SS and MDP) conducted the semistructured interviews between September 2023 and January 2024. Patients who had the opportunity to use telerehabilitation either as part of inpatient or outpatient medical rehabilitation over the last 6 months were eligible to participate. The 6-month period was chosen to avoid recollection bias. Health care professionals were invited to participate if they were involved in the development or implementation of telerehabilitation within their institutions. Recruitment was conducted through the 13 rehabilitation centers that previously participated in the quantitative surveys. Patients and health care professionals who completed the quantitative surveys and were interested in participating in interviews were invited to contact the researchers directly by email.

As recruitment had been carried out through the rehabilitation centers, the participants and researchers did not meet or know each other beforehand. There was one exception, in which one of the researchers knew a participant in medical rehabilitation aftercare; in this case, the participant was interviewed by the second researcher in the team. While planning the exploratory study, it was assumed that 20 interviews (ie, 10 per group) would achieve thematic saturation, as the interviews were based on established theoretical frameworks and the study objectives were narrowly defined [20]. Aiming for maximum variation sampling, we contacted rehabilitation centers in an attempt to recruit patients from more underrepresented diagnostic categories (eg, orthopedics). During the interviews, it became clear that thematic saturation was achieved to the extent that participants had, in general, similar positive experiences with telerehabilitation.

We conducted all interviews online via Zoom (Zoom Communications, Inc) or Webex (Cisco Systems), with no third parties present, and we used a contextualization form to systematically document the interview conditions and interactions [21]. The audio recordings were professionally transcribed.

### Data Analysis

We used qualitative content analysis based on Kuckartz and Rädiker [22] to analyze the data. The coding process was computer assisted (MAXQDA [version 2020; VERBI Software]), using a combination of deductive and inductive approaches. First, we created a codebook containing deductive categories based on the UTAUT2 and CFIR domains and anchor examples (quotations). Two researchers (SS and MDP) independently coded half of the interview material to test the categories’ plausibility, clarity, and suitability. Text passages that could not be assigned to an existing code were marked, annotated with coding suggestions, and then discussed by the 2 coders. We then merged the 2 analysis documents in MAXQDA to visualize the differences and calculate the intercoder agreement. This was not done to quantify the different codes but rather to easily locate potential problematic quotes and categories. We discussed differences, similarities, and ambiguities in the coded material to further refine and adjust the categories. In addition, inductive categories were cocreated from the comments in the previous steps. The final category system consisted of 13 main categories and 35 subcategories for the patients and 6 main categories and 98 subcategories for the health care professionals (refer to Multimedia Appendix 1 [11,12,19,23] for detailed category systems for both groups). We then analyzed all interviews using the adapted category system, collated the data, and reached consensus on the various codes using the aforementioned principle [22]. We summarized and exported the final categories and their codes to Microsoft Excel as well as illustrative quotations corresponding to each category. For the presentation of the findings, the quotations were translated from German and carefully edited for enhanced clarity. Participants who wished to give feedback (both written and oral) on the results were invited.

### Ethical Considerations

This study was approved by the Ethics Council of the University of Siegen (ER_2/2022). Prior to the interviews, all participants were informed in writing about the interview process, how their personal data would be stored, and their right to withdraw from the study and data processing at any time. All participants gave their consent to participate in the study. To ensure confidentiality and anonymity during data analysis, participants were assigned pseudonyms after the interviews were conducted, and personal data that could reveal their identity was pseudonymized. Personal data was encrypted and stored separately from the pseudonymized data for the duration of the project. In the event of revocation, only employees of the research project would have been able to reestablish a link to an individual in order to delete their data.

## Results

### Interviews With the Patients Undergoing Rehabilitation

#### Participants’ Characteristics

We interviewed 9 patients, with interviews lasting between 18 and 41 minutes. They were predominantly female (n=6, 67%) and aged between 50 and 69 years (n=6, 67%). The most frequently mentioned medical diagnosis group was neurological conditions (n=5, 56%), followed by psychosomatic (n=3, 33%) and orthopedic ailments (n=3, 33%); refer to Table 3 for participant characteristics.

**Table 3:** Sociodemographic data of patients undergoing rehabilitation (n=9).

Sociodemographic data		Patients, n (%)
**Age (y)**		
	30-39	1 (11)
	40-49	2 (22)
	50-59	3 (33)
	60-69	3 (33)
**Sex**		
	Male	3 (33)
	Female	6 (67)
**Diagnosis group (multiple answers possible)**		
	Neurology	5 (56)
	Cardiology	1 (11)
	Oncology	1 (11)
	Orthopedics	3 (33)
	Psychosomatic disorders and psychotherapy	3 (33)
	Other	2 (22)
**Highest educational level (German system)**		
	Master’s degree or equivalent educational program	4 (44)
	Vocational training	2 (22)
	Secondary school	1 (11)
	Other	2 (22)

#### Telerehabilitation Description and Terms of Use

Apps were mentioned as the most frequently used telerehabilitation technology, but web-based platforms were also used, mainly in aftercare. Telerehabilitation can usually be used for up to a year. The content mainly focused on balance, movement, and relaxation exercises as well as dietary tips. No prerequisites for using telerehabilitation were mentioned, apart from the basic requirement of owning an electronic device (eg, cell phone, tablet, or monitor) and a stable internet connection. In terms of cost coverage requirements, one had to have a positive employment prognosis for the German pension insurance to cover the costs (otherwise, patients had to cover the costs themselves).

#### Experience and Habits

None of the patients had previously used telerehabilitation, but some reported experience with other services, such as yoga via YouTube or fitness apps. One of the main challenges that the patients described was developing a consistent routine as part of their aftercare. This competed with the demands of daily life and the lack of external monitoring and accountability mechanisms (compared to the in-hospital rehabilitation process). One interviewee explained the following:

And so, if I manage it twice [a week], that is a lot. It’s just that, well,... I need to have the energy to do it because of my illness. The motivation would be there, but the energy is sometimes lacking. And then it’s difficult for me.

Patient 4

Keeping a consistent routine was important not only for personal recovery but also in relation to cost coverage, as most services had built-in progress monitoring processes (eg, badges or passwords needed to proceed to the next components) to ensure patients used them regularly. Otherwise, they would lose the coverage provided by the DRV:

The fact that this chat also reminds you and/or the therapists that you have to do something. Because otherwise the funding would be cancelled.... If you don’t do anything for a few weeks..., then the pension insurance would stop funding you. So you have to keep at it. I think... after a two-week break, then the reminder comes.

Patient 3

While routine proved challenging, habits were developed quickly, and some interviewees described how they did not need the guidance of the apps after a while because they had internalized the exercises. However, detailed instructions at the beginning of the exercise phase were considered helpful:

After doing this for months, I know the exercises I always do by heart and love most of them. But it’s good to have someone show you how to do everything. A video like this shows you how to do it.

Patient 3

#### Hedonic Motivation

Despite difficulties with routine training, about half of the patients undergoing rehabilitation in this study were highly motivated to use telerehabilitation. This was mainly due to their desire to recover and return to normal activities as quickly as possible:

I just want to get fit again as quickly as possible and be back on the tennis court.

Patient 9

#### Effort Expectancy

The usability of telerehabilitation was generally described as straightforward and uncomplicated. However, some patients highlighted their own technological affinity and considered that older or not-so-tech-savvy patients might experience difficulties. Adaptations for older patients, for example, via larger television screens, were described as useful and necessary. They also mentioned that deviations from the standardized (Monday to Friday during regular working hours) treatment plan could lead to complicated adaptation processes for the treatment facilities. Technical challenges were occasionally mentioned, such as minor difficulties with the app, an unstable internet connection, or high data volume:

It’s always difficult when you need Wi-Fi for technical things. I realized that on vacation. Well, we were on vacation for 14 days. We didn’t have Wi-Fi. And then you need a lot of data volume if you keep playing these movies. That’s why you drop it for that period of time.

Patient 6

In many cases, the support provided by the rehabilitation centers was described as helpful and reliable:

They would rather say, “As a hospital, we are on board. Take a look. It’s not rocket science.... It is designed for all age groups. See how you get on.... If you can’t manage, we’ll see how we can help you.” And that’s especially for the older generation, of course, invaluable.

Patient 1

#### Performance Expectancy

Patients perceived telerehabilitation as useful for their rehabilitation process, finding its low-threshold nature beneficial for their physical and mental well-being and overall health promotion:

For me, it completely fulfilled the purpose I wanted it for, which was to stay on track and get my physical and mental complaints under control. So it worked really well.

Patient 3

The most significant benefit mentioned was the time, location, and content flexibility. Above all, reducing travel to rehabilitation centers or long waiting times for an appointment was rated as positive:

I actually thought it was a good solution because it allowed more flexibility in terms of time.... For me,... I would have had to travel to [the rehabilitation center], which means a half-hour or 20-minute drive each time. That’s no longer necessary.

Patient 6

However, it was clear that telerehabilitation was only considered suitable for some patient groups. Participants mentioned fellow patients for whom its use was unthinkable due to older age, perceived effectiveness of professional exercise equipment, and the belief that face-to-face sessions promoted better discipline and correction of training techniques:

I also met people in rehabilitation who said, “No, if I don’t have a trainer standing opposite me and pushing me, and if I don’t have to go there in person, then I know I’m lying to myself and just getting lazy.”

Patient 3

#### Facilitating Conditions

The flexibility afforded by telerehabilitation in the context of aftercare was seen as conducive to an easy integration into everyday life. Furthermore, content flexibility made the exercises adaptable to existing equipment, health status, or personal preferences. In addition, content that was already familiar to patients from in-hospital rehabilitation enabled treatment continuity. Another facilitating aspect of telerehabilitation aftercare was behavioral monitoring by the rehabilitation centers. The patients described them as supervisory bodies that would monitor regular use and progress, helping them maintain the high motivation levels required to complete the aftercare program:

But the point of the whole process was to stabilize me again, to stabilize me as a patient again, rather than to betray myself. It was clear that [the rehabilitation facility] where I did it [rehabilitation] was acting as a kind of controlling authority.

Patient 2

Participants described the timely technical support, detailed instructions provided by the rehabilitation centers, and regular interactions with the therapists as crucial for their telerehabilitation use. Depending on the setting, technical support could be requested via the app or personal contact with therapists (eg, in case of installation and use uncertainties).

#### Social Influence

According to our participants, there was little dialogue with other patients about telerehabilitation during this time, so peer influence was minimal. Therapists served as the main source of social influence, as they suggested the use of telerehabilitation and integrated it into their patients’ care plans. In all cases, telerehabilitation was voluntary. Our participants reported that, in general, friends, acquaintances, family, and physicians reacted positively to the use of telerehabilitation and, frequently, spouses would provide support (eg, by integrating cooking and healthy eating suggestions).

#### Privacy Concerns

Surprisingly, in the German context, patients did not have any privacy concerns regarding telerehabilitation. The main reasons for this were limited personal or sensitive information needed to be disclosed, trust in health care professionals and app developers, simple design, existing privacy regulations, and professional experience:

And, let’s say, fears or that I somehow also because of data protection and such/I mean, what should happen? I mean, I sit there in front of the laptop, doing my exercises. And yes, so I have no fears or concerns.

Patient 5

#### Wishes for Improvement

The patients expressed various wishes for improvement related to both the used app itself and telerehabilitation in general: a larger device was deemed necessary for performing exercises, as patients reported difficulties with the limited size of smartphone screens. This would be especially beneficial for older patients, as would a more user-friendly interface. In addition, offering shorter and simpler *beginner* exercises could provide a more accessible introduction for some patients, and integrating verbal guidance for all exercises would allow them to focus on their movements without constantly glancing at the screen. For some, a more varied and natural voice for guiding patients through exercises, along with a presentation style that feels less artificial, would enhance engagement.

Other crucial aspects concerned the app’s communication tools, such as enhancing the chat function, integrating live video consultations, or adding a mailbox function for direct contact with therapists or to ensure the quick resolution of any technical issue. Introducing a reward or point system was suggested as a measure to maintain or boost user motivation. In their view, fostering better connections among patients undergoing rehabilitation by facilitating exchanges via the app or occasional in-person sessions at rehabilitation centers would significantly improve the overall experience. Finally, patients wished for improved cost transparency (concerning the coverage of digital services) and detailed information on extending the app’s use beyond the set 1-year period.

#### Intention to Use

All participants stated that they would be willing to use telerehabilitation again as part of their rehabilitation or aftercare if offered and feasible for their condition. However, requiring additional or special training equipment may be a reason not to use telerehabilitation:

So that you should do muscle building or something like that, for example, I would prefer to do it in the gym, on training devices.

Patient 6

### Interviews With Health Care Professionals

#### Description of the Sample

The 8 health care professionals who were interviewed were predominantly therapists (n=6, 75%) who mainly worked in neurology (n=5, 62%). All (n=8, 100%) of them were involved in the implementation of telerehabilitation. Again, the participants were mainly female, and the interview lasted between 30 and 58 minutes (refer to Table 4 for participant characteristics).

**Table 4:** Health care professionals’ sociodemographic data and telerehabilitation characteristics (n=8).

Sociodemographic data			Health care professionals, n (%)
**Age (y)**			
	20-29	1 (13)	
	30-39	3 (38)	
	40-49	3 (38)	
	50-59	1 (13)	
**Sex**			
	Male	3 (38)	
	Female	5 (62)	
**Professional position in the organization**			
	Management, administration, or leadership	3 (38)	
	Sports therapist or physiotherapist	5 (63)	
**Experience in the position (y)**			
	<1	0 (0)	
	1-4	3 (38)	
	5-10	2 (25)	
	>10	3 (38)	
**Primary treatment specialty of the institution (multiple answers possible)**			
	Cardiology	2 (25)	
	Psychosomatics and psychotherapy	2 (25)	
	Neurology	5 (63)	
	Orthopedics	3 (38)	
**Context (multiple answers possible)**			
	Outpatient rehabilitation	8 (100)	
	Inpatient rehabilitation	3 (38)	
	Prevention	3 (38)	
	Aftercare	2 (25)	
**Type of telerehabilitation (multiple answers possible)**			
	App training	6 (75)	
	Digital training courses	3 (38)	
**Year of telerehabilitation implementation**			
	2020	3 (38)	
	2021	3 (38)	
	2022	1 (13)	
	Unknown	1 (13)	

#### Characteristics of Telerehabilitation

Aftercare consists of a training program that is usually completed within a 1-year period and integrates health promotion content and physical exercises. Smartphones, tablets, or laptops are typically used as devices, though some content is also delivered via patient television during their stay at the centers. Most telerehabilitation offers have been developed either internally by the rehabilitation centers or by external developers of telerehabilitation services and were introduced during the COVID-19 pandemic between 2020 and 2022. Since then, rehabilitation centers have mainly been using apps (developed internally or by an external company), providing patients with educational resources or physical exercises that can be adapted to their particular diagnoses. Some participants also described the existence of a portal or web-based platform which can be used to obtain information and training, and, in some cases, training courses were digitized, for example, own platform or YouTube.

#### Innovation Domain

##### Relative Advantages and Disadvantages of Telerehabilitation

The most important advantages mentioned were flexibility (in terms of time and place) for both patients and health care professionals and resource efficiency within the institution, especially in terms of time and staff:

Well, the flexibility, definitely.... It is much easier to reconcile work, family, and everyday life if you can do it from home rather than having to travel to another facility for half an hour or 45 minutes.

Health care professional 3

Yes, we also use it [digital education] to prepare for seminars, for example, so that patients first watch a video with basic information and then simply deepen their existing knowledge in a mutual exchange with the other rehabilitation patients and the lecturer or therapist. It just saves time, because you don’t have to spend another 20 or 30 minutes giving a lecture. So the patients come to the appointment with the information already. And there is also the advantage that not everyone can absorb knowledge at the same speed.

Health care professional 9

Furthermore, telerehabilitation could reach more patients and meet quality standards more easily, as there was less deviation in terms of content due to the digital format:

What I believe is also an advantage, when you think again of the requirements for patient training, is that a digital offering naturally ensures that the content, structure, and sequence always remain the same. So, there are no differences, as we know them from traditional patient training. Therapist A presents it differently despite the manual. And therapist B finds the topic interesting in a different way and presents it differently. And so, in the end, you actually distort something that has been standardized.... Of course, this does not happen with telerehabilitation, because I have the structure once, and it stays.

Health care professional 5

The biggest disadvantage mentioned by some interviewees was the lack of opportunity to perform manual examinations and provide treatment (eg, by palpation) and monitor treatment progress:

What I miss in all this digital aftercare is what we have in therapy, the assessment that allows me to judge whether what I have done has changed anything after eight weeks. Because the patient telling me, “Yes please, change exercise 500 for me or exercise 459 is too hard,” that doesn’t tell me anything, right?

Health care professional 4

One other aspect that several health experts found disadvantageous was the technical barriers that often have to be overcome when using digital services:

And otherwise, there can always be technical problems. That can happen, of course. Even if it is rare for an app not to work on any patient’s devices.

Health care professional 2

##### Innovation Costs

Little was known about the rehabilitation centers’ implementation and maintenance costs, as many therapists had no insights into these processes. Some interviewees reported that using external developers of telerehabilitation services involved initial software and hardware costs and monthly license fees, which could serve as barriers:

Well, I can understand why that is a barrier, yes. Because the price of the software has to be paid in full immediately. And the providers certainly do that differently.... But in any case, as a hospital, you first have an investment in the software and then, of course, in the hardware. So, equipping a room like that is not cheap if you need several workstations,... and also the space, which must not be too small for this kind of training.

Health care professional 2

The need to expand the internet connection was also seen as a cost factor, as Wi-Fi accessibility for all patient rooms was now seen as essential:

Of course, they tried to provide the patients with good Wi-Fi, but with an app like this, you have to make sure that it works perfectly in every room. And that was a huge cost factor, especially in the old buildings, and it was a huge issue until it worked.

Health care professional 2

However, for the centers that developed their digital rehabilitation services in-house, these costs were not an issue:

So we didn’t have to go to any additional expense to hire external companies, but it actually happened in the course of our day-to-day business. And that’s why it wasn’t such a big barrier for us.

Health care professional 6

#### Inner Setting

##### Available Resources

The key challenges when telerehabilitation was introduced included concerns about the availability of essential resources, such as appropriate equipment, reliable internet connection, and time to develop digital services:

Then, of course, good internet. That was also sometimes a problem at the beginning. But that’s important too, of course. And all the equipment. Yes, and in terms of space, because of course you also need a room where you can do it all. Now that everything has settled in, it’s no longer a problem. But at the beginning, I think it was quite difficult in terms of planning, where to put what and how best to do it.

Health care professional 3

The need to train staff in the use of new technologies and to promote their acceptance was also emphasized:

Well, of course you first need employees who are willing to take part. And we have workstations for app training. Of course, a certain infrastructure had to be ensured so that training was even possible here on site. The employees had to be trained. The doctors had to be trained. That was a big step at the beginning, so a lot of extra work.

Health care professional 3

By contrast, the flexibility in terms of time and location afforded by telerehabilitation was emphasized as positive, particularly in light of the ongoing challenges of recruiting and retaining health care professionals:

And we can really compensate for therapy cancellations due to staff shortages by offering the therapies digitally.

Health care professional 6

##### Communication

Some of the health care professionals pointed out that communication processes have changed due to the use of telerehabilitation. Regular contact with patients via digital platforms facilitated ongoing support and exchange beyond in-person sessions. Furthermore, the new videoconferencing systems or employee chats facilitated the fast flow of information among staff:

And what we have noticed is that communication via chat and video calls—we use a videoconferencing system for this—works extremely well. We are light years ahead of email communication. We realized this after the first week... that we needed a chat [laughs] where we could exchange information quickly.

Health care professional 2

While most employees were satisfied with analog and digital communication, there was also some dissatisfaction with the communication processes. Some reported communication hurdles, especially when the necessary information for a good familiarization with telerehabilitation was not provided:

For a month I didn’t really have an introduction because it was all so new. And the colleagues who were already there were supposed to support me. They had only been there for two or three months. And so I found that to be a big difficulty for me.

Health care professional 4

One interviewee also reported that communication about the introduction of the new offer was top down, which was interpreted as unfavorable for the implementation process:

Well, the conflict or the problem... at the employee and clinic management level was, of course, that it wasn’t an intrinsic thing.... It wasn’t the clinic thinking, hey, we’ve got a great idea here, but rather it was homework imposed by executive management. So, there was a project plan. There was a corresponding guideline that was formulated. No one was asked beforehand.

Health care professional 5

##### Institutional Culture

The corporate culture was considered very important, and interviewees highlighted patient-centeredness as essential, as rehabilitation and aftercare should be tailored to the patients’ needs and circumstances:

Important, very, very important in this matter, we tell our patients along the way, no stress. The goal of our aftercare is to achieve positive results. And whether it’s four months, eight months or a year later.... I don’t care.

Health care professional 1

The health care professionals also mentioned the availability of diverse digital aftercare options, such as Zoom meetings, live events, and group sessions, so patients could choose the format that best suited their needs.

In general, regular dialogue and learning culture within teams were highly valued. Most of the interviewees felt it was important that staff members were involved in the introduction of new technologies and that the necessary training was provided, so that everyone could acquire the skills needed to use digital services:

And then I and other colleagues involved... discussed with them [each colleague] what we do in this introductory session with the patients. That is, to make sure that the program is clearly explained, that it is clear what will be discussed and what content will be shown.

Health care professional 7

#### Implementation Process Domain

##### Assessing Needs

Considering the needs of therapists and patients emerged as an essential aspect of the implementation process. Some interviewees described initial reluctance on the part of the staff to implement and use telerehabilitation, mainly when they had to create the video or audio content themselves. Taking these concerns and objections seriously and “guiding staff” on the journey to implementation was seen as fundamental. In addition to the needs and requirements of the staff, the participants felt that the circumstances of the patients (eg, advanced age or cognitive abilities) should also be considered. However, there were not many objections to using telerehabilitation, and the rehabilitation centers generally tried to facilitate it for all their patients.

##### Engaging Patients and Staff

Participants emphasized that therapists could only promote telerehabilitation to patients if they themselves were convinced of it and reported that implementing telerehabilitation requires particularly committed individuals. Therefore, convincing and involving the rest of the team in the implementation processes was necessary:

It was always openly discussed in the teams, and we just looked to see who was also enthusiastic about the topic. And not simply impose and say, now go and check it out for yourselves. And that was how it was implemented.

Health care professional 2

#### Individuals’ Domain

##### Capability

Interviewees came from a variety of professional backgrounds (eg, physiotherapists or sports therapists and administrators) and were involved to varying degrees in the development, implementation, and delivery of telerehabilitation. Some respondents indicated that they now work exclusively as digital therapists. Health care professionals emphasized that a certain level of affinity for technology is required on the part of patients and health care professionals to be able to use and deliver telerehabilitation.

In individual cases, patients who were less tech-savvy were excluded from telerehabilitation. According to our interviewees, language barriers (including due to aphasia) were not necessarily an obstacle, as most exercises could often be carried out despite them. Moreover, relatives sometimes supported patients who did not have the necessary skills and knowledge, to still benefit from the service. Due to these limitations and different settings, some medical professionals suggested hybrid approaches as the most appropriate solution:

But in the end, I think the hybrid approach is more important. You have to say,... where does it make sense to do something digitally, and where does it make less sense?

Health care professional 8

##### Motivation

Interviewees emphasized that staff motivation plays a vital role in implementing telerehabilitation. A certain degree of independence from the employer was also seen as an essential implementation factor. As mentioned earlier, the health experts underlined that a team needs people who drive the process forward and who are willing to take responsibility and deviate from “service by the book” when necessary:

I am the one who has the motivation to implement these projects in the company.... I try to somehow manage to keep myself motivated to implement them. And yes, it’s a difficult process. But you need one like that. Just as [laughs] you also need someone who, let’s say, keeps a Friday sports group together, right?

Health care professional 8

The patients’ motivation was also considered essential for successfully implementing telerehabilitation. As stated in the interviews with the patients undergoing rehabilitation, they were encouraged to take responsibility for their well-being and perform exercises regularly via different motivational strategies and control mechanisms, especially in the aftercare program. However, the health care professionals mainly received feedback on telerehabilitation from patients who were enthusiastic about the program; less motivated patients were more challenging to reach:

As you might expect, some think it’s great, some think it’s annoying, and some think it just belongs there, a neutral attitude. But I think it’s being handled well.... We have the ability to control it. And we plan to address it regularly here at the site, and also to work together on some of the content again, just to make it more present for the patients...we are actually getting pretty good feedback.

Health care professional 7

##### Implementation Leads and Team Members

As mentioned earlier, interviewees described the involvement of the immediate team as essential to the implementation of telerehabilitation as well as the support of other departments within the organization, such as IT, human resources, or product management. Although decisions were often made at management level, they considered it essential to involve staff members and allow them to decide the extent of their involvement in implementing telerehabilitation, for example, performing on camera:

So the decision first has to be made at management level. And then, in our case, I think it was a sensible approach, we had project teams. One central project team that always initiated and implemented the first steps at a site.

Health care professional 2

According to the interviewees, most of the telerehabilitation implementation work was done by individual employees who had been given a mandate by management. On the one hand, this allowed a certain degree of freedom, but on the other hand, this meant it took more work to get colleagues onboard with the project. Overall, the process was seen as challenging, and support from senior management was crucial:

But it is true, as I also realized at the time, that if the chief physicians don’t support it, develop it, or implement it, then, of course, you generally have problems with implementation.

Health care professional 5

##### Support

According to the health care professionals, both the rehabilitation centers and patients received support in implementing telerehabilitation or in case of technical difficulties. Therefore, the internal IT department and the external developers of telerehabilitation services were considered essential:

The support from the developer was comprehensive. I would say that we received very strong support [from them], with regular get-togethers and meetings on-site and online, which are still taking place.

Health care professional 2

When patients needed support, health care professionals were usually the first point of contact. They decided which questions they could answer themselves or in which cases IT or building services must be involved. In addition, some rehabilitation centers encouraged patients to support each other in using digital services, as highlighted by one of the interviewees:

Well, we’re open to that and then help them with it. But I also appeal to the patients in the rehabilitation groups to help each other. Because I think that’s another resource that should be used, because in my opinion patients are a team. So, they are a group that comes together again and again in different... therapies. And they get to know each other a bit over time. They should also support each other. So, if there is someone who is really good with mobile phones, they can help a patient who is a bit older and maybe doesn’t have much to do with mobile phones. I think we as therapists should encourage and demand that.

Health care professional 8

#### Outer Setting

##### Critical Incidents as Driving Factors for Implementation

Participants emphasized that the pandemic was one of the driving factors behind the introduction of telerehabilitation. External constraints, such as distance and hygiene regulations, had to be responded to quickly, and telerehabilitation provided an alternative. Otherwise, digitization would have progressed much more slowly without the pandemic:

But a driving factor was the coronavirus pandemic. [It] simply forced us to introduce digital lectures. Or rather, it was an opportunity for us to maintain the quality of the lectures and continue to offer lectures to the same extent as before and even beyond.

Health care professional 2

##### Policies and Laws

Interviewees who were able to provide information on the approval process for telerehabilitation reported that although the acceptance of telerehabilitation is growing on the part of funding bodies, the approval process still needs to be improved in some cases. Concerning funding, it was explained that DRV only funds the digital rehabilitation aftercare services but not the on-site app training at the centers, thus incurring additional costs. The health care professionals would like the payers to continue to push for digitalization and for telerehabilitation to be reimbursed at the same amount as in-person services:

I already mentioned briefly that acceptance on the part of cost bearers is really growing when it comes to digital [offers]. It’s not happening as quickly as we would have liked.

Health care professional 6

It was also requested that the same quality criteria should be applied to the implementation of telerehabilitation as for analog services (eg, rehabilitation therapy standards and patient education guidelines).

## Discussion

### Principal Findings

This study identified several barriers, including concerns among health care professionals regarding resource availability and team communication, as well as initial staff resistance to the introduction of new technologies. Similar to other countries, health care professionals noted that, despite growing acceptance among payers, improved approval procedures and proper financing of digital services are needed for the sustainable success of telerehabilitation [24].

This study also provided insights into the challenges patients faced with the routine use of telerehabilitation in aftercare. However, they reported high acceptance of telerehabilitation and expressed a wish for improved usability, for example, in terms of voice, music, and instructions.

A direct comparison of the 2 groups’ perspectives cannot be made due to the different theoretical frameworks used for each group. However, there are aspects that play a crucial role in the implementation and acceptance of telerehabilitation for both groups.

Overall, both patients and health care professionals were enthusiastic about the potential of telerehabilitation and appreciated its flexibility. For patients, this often means less travel for aftercare, while for health care professionals, the advantages relate to opportunities to work from home or to standardize certain workflows, such as training programs.

There were also similarities in terms of motivation. The patients in this study were highly motivated to use the prescribed telerehabilitation. Health care professionals confirmed this, stating that more than half of their patients were highly motivated to use telerehabilitation. In addition, rehabilitation centers valued various motivational mechanisms to maintain control over exercise performance, which patients perceived as helpful for maintaining a routine.

Another aspect that both groups found helpful was continuous, comprehensive support. Patients appreciated the prompt assistance provided by rehabilitation facilities when questions or difficulties arose, whether during in-clinic rehabilitation or aftercare. This aligned with the participating clinics’ patient-centered values and therapists’ emphasis on providing tailored support and exercises. Fast and continuous technical support, for example, from developers or IT departments, was also identified as essential for successful implementation.

Similar views were expressed regarding obstacles and concerns. Both groups pointed to occasional technical problems and more comprehensive structural issues, such as poor internet connections. They also mentioned that telerehabilitation is not an option for people who lack technical affinity or who are concerned that they would not be able to participate in digital programs reliably.

Both groups also mentioned that telerehabilitation is not a suitable sole therapy or aftercare option. For certain diagnoses and groups of people, it is important to provide an analog service; therefore, a hybrid service is a sensible solution. In general, health care professionals seem to be somewhat more skeptical of telerehabilitation than patients. They tended to express concerns about internal organizational processes and associated costs.

Our findings largely confirm the results of other studies and suggest that the insights gained in the international context regarding acceptance and implementation conditions can also be transferred to the German context. Overall, telerehabilitation was well accepted by patients, with all of them stating that they would use it again if the necessary conditions were met. The high level of acceptance among telerehabilitation users is consistent with other studies based on the UTAUT2 framework [9,25-27]. Previous studies have also identified support, particularly with regard to technology [28,29], patient motivation [30,31], and the advantages of telerehabilitation, such as flexibility [32,33], as factors that contribute to acceptance and facilitate implementation. However, challenges arise primarily from patients’ varying levels of familiarity with technology and difficulties in establishing routines.

From the perspective of health care professionals, the most important facilitators were the perceived benefits of flexibility, resource efficiency, and improved communication with patients. Some authors also point out the flexibility for both sides as beneficial [34], while others fear that the use of telerehabilitation could also lead to a reduction in the effort required to communicate with patients [35]. However, challenges arise from the varying technological affinities among patients, which can hinder the adoption of telerehabilitation and require tailored support for those less familiar with digital tools [2].

However, this study also points to some divergent findings. Other studies show that *privacy concerns* can play an important role in the acceptance of digital rehabilitation services [36], which is why we expanded the UTAUT2 model. Nevertheless, all interviewees assured us that they had no *privacy concerns*.

In addition, other studies suggest that the limited knowledge of health care professionals regarding the handling and implementation processes is one of the main barriers to the introduction of telerehabilitation [2]. This cannot be fully confirmed by our interviews. Although there were requests for more in-depth training on the part of health care professionals, respondents did not generally indicate that they felt overwhelmed by the new technologies. The lack of technical skills was clearly seen as more of a problem for patients than for health care professionals.

### Methodological Strengths and Limitations

Due to its qualitative approach, this study provides in-depth insights into the conditions for implementing and accepting telerehabilitation. Our methodology is based on 2 well-established theories [12,19] that have already been applied in previous studies. This facilitates the comparability of results with other research findings.

However, the predominantly positive results of this study may be due to selection bias, limited access to the field, and a preferential participation of patients and health care professionals who value digitalization. Despite intensive efforts to recruit additional interview participants, only 9 interviews with patients could be conducted instead of the planned 10. It can be assumed that patients who were motivated and satisfied with telerehabilitation were more willing to discuss their experiences. Future studies should also survey patients who discontinued or refused telerehabilitation. Our efforts to recruit such participants, for example, through patient groups and social media, were unsuccessful, so our findings do not reflect alternative perspectives.

Recruitment was also the most significant limitation regarding health care professionals. Of the 10 planned interviews with health care professionals, 8 (80%) could be conducted, mainly via rehabilitation centers that had already participated in the quantitative surveys, valued telerehabilitation, and had a narrow clinical focus (ie, mainly neurology). Despite our intensive efforts and renewed contact with the centers, we were unable to acquire additional interview partners within the time frame of this study.

Accordingly, this study mainly details the experiences and attitudes of neurology clinics and patients who were also predominantly female, more engaged, more tech-savvy, and more willing to participate in the survey than people who are reluctant to use telerehabilitation. All these aspects may limit the generalizability of the results.

### Conclusions

In this study, both patients and health care professionals viewed telerehabilitation as beneficial. They particularly appreciated the flexibility and accessibility it offered. Patients demonstrated a high level of acceptance and willingness to continue telerehabilitation, citing expected health benefits and supportive environments as critical facilitators. However, challenges, such as varying technological familiarity and routine establishment, were mentioned as potential barriers to continued participation. The implementation of telerehabilitation in rehabilitation centers was influenced by available resources and the engagement and support of all health care professionals.

In light of these results, we recommend a hybrid model that combines digital solutions with traditional in-person therapy as well as increased patient-to-patient exchange and support. In addition, appropriate structural and financial conditions are needed to integrate telerehabilitation into medical rehabilitation care. This includes streamlining approval processes and ensuring equitable funding comparable to traditional rehabilitation methods to enable broader access and sustainable implementation across various health care settings.

## Appendix

Multimedia Appendix 1 Category systems.

## Abbreviations

CFIR: Consolidated Framework for Implementation Research
DRV: Deutsche Rentenversicherung (German Pension Insurance Association)
UTAUT2: extended unified theory of acceptance and use of technology
