# Supplementary material for: Investigating the Acceptance and Implementation Conditions of Telerehabilitation in Germany Among Patients and Health Care Professionals: Qualitative Interview Study
Source: JMIR Rehabil Assist Technol. 2025 Aug 1;12:e68766. doi: 10.2196/68766 (PMC12357121; doi:10.2196/68766)
Supplement: Multimedia Appendix 1 [file rehab_v12i1e68766_app1.docx]

| **Coding categories for patient interviews according to UTAUT** (UTAUT dimensions according to Venkatesh [11,12]; expanded and adapted to the project context.) | | |
| --- | --- | --- |
| **Coding categories** | | **Definitions** |
| **Rehabilitation in general** | | Issues relating to the rehabilitation process in general. |
|  | Focus of rehabilitation | Diagnosis category and/or treatment focus of rehabilitation. |
|  | Obstacles in getting (digital) rehabilitation | Obstacles to the access and utilization of telerehabilitation. |
|  | Experiences in general | General experiences during rehabilitation, including difficulties. |
|  | Options for selecting a telerehabilitation program | The option to choose a specific telerehabilitation program from several offers. |
| **Telerehabilitation regulations** | | Requirements that are relevant at organizational, cost-bearer or legal level for the use of telerehabilitation. |
|  | Conditions of Use | Conditions formulated by the rehabilitation center for the use of telerehabilitation. |
|  | Financing of telerehabilitation | Aspects relating to the assumption of costs for telerehabilitation, e.g. financing by the DRV, other social insurance providers or self-payers. |
| **characteristics of telerehabilitation** | | Characteristics of telerehabilitation in general. |
|  | Total duration of telerehabilitation use | Total duration in months/weeks of telerehabilitation use during medical rehabilitation. |
|  | Type of telerehabilitation | The type of telerehabilitation used by patients, e.g. an app or an internet platform. |
|  | Description of telerehabilitation | The way in which telerehabilitation is performed and the exercises it consists of. |
|  | Frequency of use | Average frequency of use of telerehabilitation. |
|  | Duration of use during a session | The duration of telerehabilitation use during a session. |
|  | Start of telerehabilitation use | Date on which the use of telerehabilitation has started. |
|  | Necessary devices | Devices needed for the use of telerehabilitation. |
| **Hedonic Motivation** | | Hedonic motivation is defined as the fun or pleasure derived from using a technology. |
| **Experience and Habit** | | Experience reflects an opportunity to use telerehabilitation or similar digital offers and is typically operationalized as the passage of time from the initial use of a technology by an individual. Habit can be seen as a result of prior performance or the extent to which people tend to act automatically because of learning. |
|  | Challenges in establishing a routine | Challenges regarding compliance with telerehabilitation as part of rehabilitation aftercare. |
|  | Previous experience | Experience already gained with telerehabilitation in the medical field before their current rehabilitation. |
| **Privacy concerns** | | The fear that your own data will not be adequately protected. |
| **Facilitation Conditions** | | Facilitating conditions are defined as the degree to which an individual believes that an organizational and technical infrastructure exists to support use of telerehabilitation. |
|  | Adaptability of telerehabilitation to personal context/preference | The degree to which telerehabilitation is adaptable to patients’ diagnosis or circumstances. |
|  | Compatibility | The degree to which telerehabilitation is perceived as being consistent with existing values, needs, and experiences of potential adopters. |
|  | Regular exchange with therapists | The degree of exchange with therapists during the use of telerehabilitation. |
|  | Support with technical difficulties | The degree of support offered when technical difficulties or questions arise. |
|  | Inclusion/participation of patients in the implementation of telerehabilitation | The degree of involvement and feedback in the implementation processes of telerehabilitation. |
|  | Introduction and training | The nature and process of the introductory and further training programs regarding telerehabilitation. |
|  | Perceived Behavioral Control | Reflects perceptions of internal and external constraints on behavior and encompasses self-efficacy, resource facilitating conditions, and technology facilitating conditions. |
| **Social Influence** | | Social influence is defined as the degree to which an individual perceives that important others believe that they should use telerehabilitation. |
| **Effort Expectancy** | | Effort expectancy is defined as the degree of ease associated with the use of telerehabilitation. |
|  | Challenges using telerehabilitation | Challenges experienced when using telerehabilitation (e.g., technical, usability, etc.). |
|  | Ease of Use | The degree to which the use of telerehabilitation is perceived as difficult. |
|  | Complexity | The degree to which telerehabilitation is perceived as relatively difficult to understand and use. |
|  | Use by older and/or non-tech-savvy patients | The assumption that older and/or non-tech-savvy patients would not use the service or would be overwhelmed by its use. |
| **Performance Expectancy** | | Performance expectancy is defined as the degree to which an individual believes that using telerehabilitation will help them. |
|  | Relative Advantage | The degree to which the use of telerehabilitation is perceived as being better than the use of analog rehabilitation. |
| Perceived Usefulness | | The degree to which a person believes that using telerehabilitation would enhance well-being. |
| **Patients' preference for in-person rehabilitation** | | The patients prefer face-to-face rehabilitation for various reasons. |
| **Improvement wishes** | | Wishes expressed by patients to optimize telerehabilitation or to create new digital services. |
| Improvement wishes - usability | | Wishes concerning the usability of telerehabilitation. |
| Improvement wishes - Human interaction | | Wishes concerning the human interaction process of telerehabilitation. |
| Improvement wishes - reduced complexity | | Wishes concerning the complexity of telerehabilitation. |
| Improvement wishes - functional variability | | Wishes concerning the functional variability of telerehabilitation. |
| Improvement wishes - info on compatible devices | | Wishes for information on the compatibility of telerehabilitation. |
| Improvement wishes - info on financing telerehabilitation | | Wishes for information about the costs of telerehabilitation. |
| **Intention to use/Behavioral intention** | | Behavioral intention refers to the subjective probability that a person will perform a specified behavior, i.e., use telerehabilitation [23] |
| Satisfaction with telerehabilitation | | The general satisfaction with telerehabilitation. |

| **Coding categories for healthcare professionals’ interviews according to CFIR** (CFIR dimensions according to Damschroder et al. [19] expanded and adapted to the project context.) | | | | |
| --- | --- | --- | --- | --- |
| **Coding categories** | | | | **Definitions** |
| **CHARACTERISTICS OF TELEREHABILITATION** | | | |  |
|  | Description of telerehabilitation | | | The way in which telerehabilitation is performed and the exercises it consists of. |
|  | Duration of use | | | The prescribed duration of telerehabilitation use. |
|  | Date of introduction | | | The date or period when telerehabilitation was introduced. |
|  | Proportion of patients who use telerehabilitation | | | The (percentage) share of rehabilitants who are offered telerehabilitation or who use telerehabilitation. |
|  | Type of telerehabilitation | | | Type of telerehabilitation offered by the rehabilitation centers, e.g. an app or an internet platform. |
| **INNOVATION DOMAIN** | | | | The “thing” being implemented, e.g., a new clinical treatment, educational program, or city service. |
|  | Innovation Source | | | The group that developed and/or visibly sponsored use of telerehabilitation is reputable, credible, and/or trustable. |
|  | Innovation Evidence-Base/Quality | | | Telerehabilitation has robust evidence supporting its effectiveness. |
|  | Innovation Relative Advantage | | | Telerehabilitation is better than other available innovations or current practice. |
|  | Innovation disadvantages | | | Disadvantages of telerehabilitation compared to status quo. |
|  | Innovation Adaptability | | | Telerehabilitation can be modified, tailored, or refined to fit local context or needs. |
|  | Innovation Complexity | | | Telerehabilitation is complicated, which may be reflected by its scope and/or the nature and number of connections and steps. |
|  | Innovation Design | | | Telerehabilitation is well designed and packaged, including how it is assembled, bundled, and presented. |
|  | Innovation Cost | | | Telerehabilitation purchase and operating costs are affordable. |
|  | Conditions of use | | | Conditions formulated by the rehabilitation center for the use of telerehabilitation. |
|  | Improvement wishes | | | Wishes to improve different aspects of implemented telerehabilitation. |
|  |  | *Increased use of telerehabilitation in aftercare* | | Wishes concerning the wider use and acceptance of telerehabilitation in rehabilitation aftercare. |
|  |  | *Increased acceptance among cost bearers* | | Wishes concerning the wider acceptance and support of telerehabilitation among cost bearers. |
|  |  | *Usability* | | Wishes concerning the usability of telerehabilitation. |
|  |  | *Human interaction* | | Wishes concerning the human interaction process of telerehabilitation |
|  |  | *Reduced complexity* | | Wishes concerning the complexity of telerehabilitation. |
|  |  | *Functional variability* | | Wishes concerning the functional variability of telerehabilitation. |
|  |  | *Info on compatible devices* | | Wishes for information on the compatibility of telerehabilitation. |
|  |  | *Info about financing of telerehabilitation* | | Wishes for information on telerehabilitation costs. |
| **OUTER SETTING DOMAIN** | | | | The setting in which the Inner Setting exists, e.g., hospital system, school district, state. There may be multiple Outer Settings and/or multiple levels within the Outer Setting (e.g., community, system, state). |
|  | Critical Incidents | | | Large-scale and/or unanticipated events disrupt implementation and/or delivery of telerehabilitation. |
|  | Local Conditions | | | Economic, environmental, political, and/or technological conditions enable the Outer Setting to support implementation and/or delivery of telerehabilitation. |
|  | Data protection concerns | | | Concerns regarding data protection and steps needed to address this. |
|  | Partnerships & Connections | | | The Inner Setting is networked with external entities, including referral networks, academic affiliations, and professional organization networks. |
|  | Policies & Laws | | | Legislation, regulations, professional group guidelines and recommendations, or accreditation standards support implementation and/or delivery of telerehabilitation. |
|  | Financing | | | Funding from external entities (e.g., grants, reimbursement) is available to implement and/or deliver telerehabilitation. |
|  | External Pressure | | | External pressures drive implementation and/or delivery of telerehabilitation. |
| **INNER SETTING DOMAIN** | | | | The setting in which telerehabilitation is implemented, e.g., rehabilitation center. There may be multiple Inner Settings and/or multiple levels within the Inner Setting. |
|  | Institutional rehabilitation focus | | | The medical specialty of the rehabilitation center. |
|  | Previous experience with telerehabilitation | | | Health professionals' previous experience with telerehabilitation in their work. |
|  | Expectations of telerehabilitation | | | Institution's expectations for the implemented or developed telerehabilitation. |
|  | Reasons for implementation | | | Specific reasons why the institution implemented telerehabilitation (not covered by Outer Domain codes). |
|  | Physical Infrastructure | | | Layout and configuration of space and other tangible material features support functional performance of the Inner Setting. |
|  | Technical Infrastructure | | | Technological systems for telecommunication, electronic documentation, and data storage, management, reporting, and analysis support functional performance of the Inner Setting. |
|  | Work Infrastructure | | | Organization of tasks and responsibilities within and between Individuals and teams, and general staffing levels, support functional performance of the Inner Setting. |
|  | Relational Connections | | | There are high quality formal and informal relationships, networks, and teams within and across Inner Setting boundaries (e.g., structural, professional). |
|  | Communication | | | There are high quality formal and informal information sharing practices within and across Inner Setting boundaries (e.g., structural, professional). |
|  | Institutional Culture | | | There are shared values, beliefs, and norms across the Inner Setting. |
|  |  | *Human Equality-Centeredness* | | There are shared values, beliefs, and norms about the inherent equal worth and value of all human beings. |
|  |  | *Recipient-Centeredness* | | There are shared values, beliefs, and norms around caring, supporting, and addressing the needs and welfare of recipients. |
|  |  |  | *Patient satisfaction* | Patients' satisfaction is pursued and assessed (e.g., through feedback). |
|  |  |  | *Providing choices to patients* | Patients are presented options in the telerehabilitation implementation/use. |
|  |  |  | *Addressing patient barriers* | Potential barriers to the use of telerehabilitation by patients are addressed/prevented at institutional level. |
|  |  |  | *Challenges in establishing a routine* | Patients' challenges in using telerehabilitation frequently/as prescribed. |
|  |  | *Deliverer-Centeredness* | | There are shared values, beliefs, and norms around caring, supporting, and addressing the needs and welfare of deliverers. |
|  |  | *Learning-Centeredness* | | There are shared values, beliefs, and norms around psychological safety, continual improvement, and using data to inform practice. |
|  | Tension for Change | | | The current situation is intolerable and needs to change. |
|  | Compatibility | | | Telerehabilitation fits with workflows, systems, and processes. |
|  | Relative Priority | | | Implementing and delivering telerehabilitation is important compared to other initiatives. |
|  | Incentive Systems | | | Tangible and/or intangible incentives and rewards and/or disincentives and punishments support implementation and delivery of telerehabilitation. |
|  | Mission Alignment | | | Implementing and delivering telerehabilitation is in line with the overarching commitment, purpose, or goals in the Inner Setting. |
|  | Available Resources | | | Resources are available to implement and deliver telerehabilitation. |
|  |  | *Personnel* | | Staff is available for the implementation of telerehabilitation. |
|  |  | *Time* | | Time is available for the implementation of telerehabilitation. |
|  |  | *Funding* | | Funding is available to implement and deliver telerehabilitation. |
|  |  | *Space* | | Physical space is available to implement and deliver telerehabilitation. |
|  |  | *Technical Equipment* | | Required technical equipment for telerehabilitation |
|  |  | *Materials & Equipment* | | Supplies are available to implement and deliver telerehabilitation. |
|  |  | *Access to Knowledge & Information* | | Guidance and/or training is accessible to implement and deliver telerehabilitation. |
|  |  |  | *Training and introduction* | The nature and process of the introductory and further training programs in the field of telerehabilitation for patients. |
| **INDIVIDUALS DOMAIN** | | | | The roles and characteristics of Individuals involved in the implementation process. |
|  | **Roles Subdomain** | | | Project Roles |
|  |  | *Leader/Manager* | | Individuals with a high level of authority, including key decision-makers, executive leaders, directors, leaders with the authority to dedicate resources and to make decisions about whether to adopt, implement, and or/sustain telerehabilitation. |
|  |  | *Implementation Leads* | | Individuals who lead efforts to implement telerehabilitation. |
|  |  | *Implementation Team Members* | | Individuals who collaborate with and support the Implementation leads to implement telerehabilitation, ideally including telerehabilitation deliverers and recipients. |
|  |  | *Other Implementation Support* | | Individuals who support the Implementation leads and/or implementation team members to implement telerehabilitation. |
|  |  |  | *Support from family members* | Support that rehabilitants receive from their family members when using telerehabilitation. |
|  |  | *Innovation Recipients* | | Individuals who are directly or indirectly receiving telerehabilitation. |
|  |  | Innovation Deliverers | | Individuals who are directly or indirectly delivering telerehabilitation. |
|  |  | Implementation Facilitators | | Individuals with subject matter expertise who assist, coach, or support implementation. |
|  | **Characteristics subdomain** | | | Project characteristics |
|  |  | *Need* | | The indiviual has deficits related to survival, well-being, or personal fulfillment, which will be addressed by implementation and/or delivery of telerehabilitation. |
|  |  | *Capability* | | The individual has interpersonal competence, knowledge, and skills to fulfill role. |
|  |  | *Opportunity* | | The individual has availability, scope, and power to fulfill role. |
|  |  | *Motivation* | | The Individual is committed to fulfilling Role. |
|  |  |  | *Innovation deliverers* | Issues around the motivation to use/implement telerehabilitation among health professionals. |
|  |  |  | *Innovation recipients* | Issues around the motivation to use/implement telerehabilitation among service patients. |
| **IMPLEMENTATION PROCESS DOMAIN** | | | | The activities and strategies used to implement telerehabilitation. |
|  | Teaming | | | Working together, intentionally coordinating and collaborating on interdependent tasks, to implement telerehabilitation. |
|  | Assessing Needs | | | Collect information about priorities, preferences, and needs of people. |
|  |  | *Innovation Deliverers* | | Collect information about the priorities, preferences, and needs of deliverers to guide implementation and delivery of telerehabilitation. |
|  |  |  | *Satisfaction with telerehabilitation* | Satisfaction among staff of telerehabilitation - after use. |
|  |  |  | *Concerns on the part of staff* | Concerns expressed by staff regarding telerehabilitation. |
|  |  |  | *Staff attitudes towards telerehabilitation* | General attitudes and acceptance of telerehabilitation among health professionals (before and during the implementation of telerehabilitation). |
|  |  | *Innovation Recipients* | | Collect information about the priorities, preferences, and needs of recipients to guide implementation and delivery of telerehabilitation. |
|  |  |  | *Groups that cannot benefit from telerehabilitation* | User groups that due to different characteristics/barriers cannot use or benefit from telerehabilitation as intended. |
|  |  |  | *Reasons against the use of telerehabilitation* | Patients' reasons against the use of telerehabilitation. |
|  |  |  | *Preference for in-person rehabilitation* | The rehabilitants prefer face-to-face rehabilitation for various reasons. |
|  | Assessing Context | | | Collect information to identify and appraise barriers and facilitators to implementation and delivery of telerehabilitation. |
|  | Planning | | | Identify roles and responsibilities, outline specific steps and milestones, and define goals and measures for implementation success in advance. |
|  | Tailoring Strategies | | | Choose and operationalize implementation strategies to address barriers, leverage facilitators, and fit context. |
|  | Engaging | | | Attract and encourage participation in implementation and/or telerehabilitation. |
|  |  | *Innovation Deliverers* | | Attract and encourage deliverers to serve on the implementation team and/or deliver telerehabilitation. |
|  |  | *Innovation Recipients* | | Attract and encourage recipients to serve on the implementation team and/or participate in telerehabilitation. |
|  | Reflecting & Evaluating | | | Collect and discuss quantitative and qualitative information about the success of implementation and/or telerehabilitation |
|  |  | *Implementation* | | Collect and discuss quantitative and qualitive information about the success of implementation. |
|  |  | *Innovation* | | Collect and discuss quantitative and qualitative information about the success of telerehabilitation. |
|  | Adapting | | | Modify telerehabilitation and/or the Inner Setting for optimal fit and integration into work processes. |
